# Paralysie flasque en début de grossesse: penser à l'hypokaliémie due aux vomissements gravidiques, à propos de deux observations dans un pays en voie de développement

**DOI:** 10.11604/pamj.2015.21.119.6867

**Published:** 2015-06-12

**Authors:** Maouly Fall

**Affiliations:** 1 Service de Médecine Interne CHN de Pikine, Sénégal

**Keywords:** Paralysie flasque, vomissement gravidique, hypokaliémie, flaccid paralysis, emesis gravidarum, hypokalemia

## Abstract

Les vomissements gravidiques peuvent être responsables de rares complications neuromusculaires mais graves notamment la paralysie hypokaliémique. Nous rapportons des observations de deux jeunes femmes d'origine guinéenne, sans histoires familiales ni antécédents particuliers, admises pour paralysie flasque des quatre membres dans un contexte de vomissements incoercibles en début de grossesse. Le bilan retrouvait une hypokaliémie majeure associée à quelques troubles électro-cardiographiques. L'apport de potassium par voie parentérale avait permis une récupération motrice totale. La paralysie hypokaliémique est une complication rare des vomissements gravidiques. Une supplémentation potassique prudente avec une surveillance électro-cardiographique et biologique permet une disparition spectaculaire des troubles neuromusculaires.

## Introduction

Les hypokaliémies sont des causes rares de paralysie musculaire. Lorsqu'elles sont sévères elles peuvent rapidement entrainer une atteinte musculaire grave qui peut même toucher les muscles respiratoires entrainant ainsi un risque létal. Les vomissements gravidiques peuvent en être l'origine. Nous rapportons 2 observations de jeunes femmes d'origine guinéenne, toutes admises au service de Médecine Interne du Centre Hospitalier National de Pikine à Dakar (Sénégal) pour déficit moteur flasque survenu dans un contexte d'aménorrhée due à une grossesse.

## Patient et observation

Mme M S S âgée de 25 ans, sans antécédents particuliers personnels ou familiaux connus, quatrième geste, troisième pare dont 3 enfants vivants bien portants, qui présentait une semaine avant son admission, dans un contexte d'aménorrhée 5 mois avec des vomissements incoercibles et algies diffuses, un syndrome neurogène périphérique des 4 membres avec une quadriparèsie cotée à 1/5 aux membres inférieurs et 2/5 aux membres supérieurs, des réflexes ostéo-tendineux abolis, une hypotonie généralisée. Il y avait également une hyperglycémie pure à 1,72g/L. Par ailleurs les constantes hémodynamiques étaient bonnes avec une pression artérielle à 110/80mmHg, une température à 36°, un pouls à 100 battements par minute, une fréquence respiratoire à 16 cycles par minute. La conscience était claire avec un score de Glasgow à 15. Il n'y avait pas de troubles sensitifs. Les réflexes cutanéo-plantaires étaient en flexion. L'examen des nerfs crâniens était normal. Il n'y avait pas de signes méningés ni de troubles trophiques. La respiration était calme et régulière et la saturation à l'aire ambiante à 98%. La diurèse était conservée. L’échographie obstétricale objectivait une grossesse évolutive d'environ 22 semaines d'aménorrhée. La radiographie pulmonaire était normale. L’électrocardiogramme montrait un sous-décalage du segment ST. Il y avait une anémie normochrome microcytaire à 10,5g/dL. Les enzymes musculaires (CPK et LDH) étaient normales de même que les transaminases. La fonction rénale était conservée. L'ionogramme sanguin était perturbé avec une hypokaliémie à 2mmol/L, une natrémie normale à 138mmol/L et une chlorémie à 85mmol/L. Le bilan phosphocalcique et les enzymes thyroïdiennes et l’électroneuromyogramme (fait à distance) étaient normaux. Cependant l'ionogramme urinaire, la gazométrie et le dosage des bicarbonates n’étaient pas réalisés de même que le bilan génétique. Ainsi nous évoquions le diagnostic d'une paralysie hypokaliémique due aux vomissements gravidiques. Le traitement consistait à une supplémentation potassique par du chlorure de potassium en raison de 1g/heure au pousse-seringue électrique à travers un cathéter jugulaire pendant 5 heures sur 2 jours. Nous en associons une réhydratation par du sérum physiologique et un traitement antiémétique par du métopimazine. L’électrocardiogramme réalisé bi-quotidiennement était devenu normal. La glycémie était devenue normale après 2 jours de réhydratation. A j + 3 nous notions une bonne évolution clinique avec une récupération motrice de la patiente qui commençait à se déplacer toute seule. A j + 4 elle sortait de l'hôpital avec un traitement per os potassique, martial et antiémétique avec une demande de consultation en gynécologie. A j + 21 elle revenait en consultation neurologique avec un ionogramme sanguin normal sans signe neurologique particulier.

Mme D.B âgée de 20 ans primigeste nullipare sans antécédents particuliers signalés qui était admise pour paralysie des 4 membres sur grossesse d'environ 4 mois suivie en gynécologie. Son tableau clinique évoluait depuis 2 semaines et s'installait de manière progressive dans un contexte de vomissements gravidiques rebelles aux traitements habituels avec asthénie et myalgies. L'examen à l'admission retrouvait un état général conservé, une pression artérielle à 100/70mmHg, une température à 37°4, un pouls à 98 battements par minute, une fréquence respiratoire à 24 cycles par minute, une saturation pulsée à 96% en aire. La conscience était claire avec un score de Glasgow à 15 mais nous notions une patiente anxieuse pour son état. Il y avait une quadriparèsie à prédominance proximal cotée à 3/5 aux 4 membres avec une hypotonie généralisée sans troubles trophiques. Les réflexes ostéo-tendineux étaient diminués et les réflexes cutanéo-plantaires en flexion. Il n'y avait pas de trouble de la sensibilité. La respiration était un peu rapide mais quand même régulière et il y avait une bonne diurèse. Le reste de l'examen était sans particularité. L’échographie obstétricale montrait une grossesse viable de 18 semaines d'aménorrhée. La radiographie des poumons était normale et l’électrocardiogramme montrait un aplatissement de l'onde T. L'ionogramme sanguin retrouvait une hypokaliémie à 2,5mmol/L, une natrémie à 140mmol/L, une chlorémie à 88mmol/L. Les enzymes musculaire et thyroïdienne, de même que la numération globulaire étaient normales. Il n'y avait aucune histoire familiale particulière notée cependant l'analyse génétique n’était pas faite et l'ENMG réalisé plutard était normal. Sous traitement à base de chlorure de potassium par voie parentérale (pousse-seringue électrique avec cathéter fémoral) en raison de 1g/heure pendant 8 heures. A cela nous associons une réhydratation et un traitement antiémétique. Douze heures après le début du traitement nous notions un début d'amélioration clinique et biologique avec une récupération sur le plan moteur et une kaliémie à 3,8 mmol/L. Le lendemain nous notions un amendement des vomissements, une récupération à 100% sur le plan moteur avec une kaliémie à 4mmol/L et un électrocardiogramme normal. Il s'agissait donc d'une paralysie hypokaliémique suite à des vomissements incoercibles sur une grossesse de 16 semaines d'aménorrhée.

## Discussion

Les vomissements gravidiques sont une affection grave qui intéresse 0,5% des femmes enceintes. Ils représentent la première cause d'hospitalisation en début de grossesse. Leur étiologie reste inconnue, mais certains auteurs suggèrent un support génétique [1]. Quand ils sont répétés, les conséquences peuvent être multiples et graves allant de la déshydratation extracellulaire associée à une hyperhydratation intracellulaire avec troubles hydro électrolytiques majeurs à l'encéphalopathie de Gayet Wernicke et la myélinolyse centropontine en passant par les complications cardiaques et musculaires striées. L'atteinte musculaire striée liée à l'hypokaliémie est rare au cours des vomissements gravidiques; elle doit donc faire rechercher d'autres causes. Elle peut être primaire, associée à un désordre génétique dans le cadre de la paralysie périodique hypokaliémique, la grossesse et l'accouchement étant des situations à risque de décompensation. La paralysie périodique hypokaliémique est une forme rare d'hypokaliémie présentant quatre formes cliniques différentes [2]: la paralysie périodique thyrotoxique hypokaliémique associant une hyperthyroïdie à une paralysie périodique; elle atteint le plus souvent des patients d'origine asiatique de sexe masculin; la deuxième forme est l'hypernatrémie paralysie périodique hypokaliémique. C'est une forme très rare associant une hypernatrémie à une paralysie périodique (une tumeur au cerveau ou une cause tuberculeuse sont le plus souvent associées à cette forme de paralysie périodique thyrotoxique hypokaliémique); une autre forme est la paralysie périodique familiale sans notion d'hyperthyroïdie ou d'hypernatrémie, très rare, et plus souvent rencontrée en Europe; enfin, la paralysie périodique sporadique ou idiopathique est un peu plus fréquente. Dans cette forme, il n'y a ni notion familiale de paralysie, ni hyperthyroïdie, ni hypernatrémie. La paralysie hypokaliémique peut être secondaire à une hypokaliémie de transfert, à des pertes rénales, à des pertes digestives telles dans le cas de nos patientes par vomissements gravidiques incoercibles. Aussi pourrait-il s'agir chez nos patientes d'une paralysie périodique sporadique ou idiopathique.

En effet la survenue de paralysies franches au cours des hypokaliémies de déplétion est rare. Sur 406 malades ayant une kaliémie inférieure à 3mmol/L, du fait de pertes rénales ou digestives, 14 cas de paralysies ont été rapportés, soit 3,4% [3]. Les simples accès de faiblesse musculaire sont, en revanche, plus fréquents. Comme dans nos cas rapportés, les paralysies des hypokaliémies par déplétion sont souvent précédées d'une période prémonitoire d'asthénie et de douleurs musculaires. Celles-ci sont susceptibles de s'aggraver à l'occasion d'une mesure thérapeutique majorant l'hypokaliémie ou à l'absence de traitement. Le tableau typique des paralysies d'origine hypokaliémique se résume en une quadriplégie flasque, symétrique, ascendante, débutant et prédominant à la racine des membres. Les réflexes tendineux et idiomusculaires sont diminués ou abolis [3]. On note l'absence de troubles sensitifs objectifs et la discrétion des troubles subjectifs. Nous avons retrouvé dans la littérature seuls deux cas de myopathies hypokaliémiques documentés dues aux vomissement gravidiques en 1983 et 2009 [4, 5]. D'autres étiologies ont été plus fréquemment citées notamment la géophagie [6], la nécrose et la résection du grêle [3], le mésentère commun [7], la mutation génétique CACNA1S [8], l'hyperthyroïdie [8, 9], l'administration de bêtamimétiques [10]. Dans les différents cas décrits, le principal point commun était une déperdition du potassium même si le mécanisme est différent et les tableaux cliniques étaient identiques. Chez nos deux patientes devant le tableau clinique dans un contexte de vomissement gravidique, l'absence d'histoire familiale particulière, les données paracliniques dont nous disposons et l’évolution favorable sous supplémentation potassique nous sommes en mesure d'affirmer notre diagnostic. Cependant vue nos conditions de pays en voie de développement nous n’étions pas en mesure de réaliser l’étude génétique de même l'ionogramme urinaire. Ce qui pourrait nous permettre de mieux asseoir le diagnostic étiologique.

## Conclusion

Les paralysies musculaires hypokaliémiques demeurent rares mais restent très grave car pouvant en jeu aussi bien le pronostic fonctionnel que le pronostic vital. Elles doivent être connues du clinicien et leur recherche s'impose devant tout contexte clinique évocateur de paralysie sur terrain de grossesse avec vomissements. L'hospitalisation devient urgente avec des bilans biologiques et électrique complet et un traitement parentéral précoce et adapté pour une meilleure préservation de la santé mère-enfant.
